# Unwilling or Unable? The Impact of Role Clarity and Job Competence on Frontline Employees’ Taking Charge Behaviors in Hospitality Industry

**DOI:** 10.3390/bs15040526

**Published:** 2025-04-14

**Authors:** Mengfen Lan, Zhehua Hu, Ting Nie

**Affiliations:** 1 School of Business, Macau University of Science and Technology, Macau 999078, China; 3240001287@student.must.edu.mo; 2 Faculty of Business and Management, BNU-HKBU United International College, Zhuhai 519087, China; zhehuahu@uic.edu.cn

**Keywords:** role clarity, job competence, organization-based self-esteem, taking charge behavior, supervisor developmental feedback

## Abstract

Hotels expect front-line staff to demonstrate greater flexibility and proactively take on more responsibility beyond their job duties, which helps to provide better customer service in an environment of uncertainty and change. Accordingly, employees’ taking charge behaviors have received widespread attention in academia and practice. Through a three-wave online survey of 352 front-line employees and their supervisors from 13 high-star hotels in the Greater Bay Area of China, this study examined the influence mechanisms of role clarity and job competence on the employees’ taking charge behavior and the moderating effect of supervisor developmental feedback. The findings indicate that frontline employees’ role clarity and job competence can enhance taking charge behavior by increasing their organization-based self-esteem. It empirically validates Proactive Motivation Theory and clarifies that employees’ proactive engagement in extra-role responsibilities depends not only on their willingness but also on sufficient competence and a clear understanding of their job roles. Supervisor developmental feedback is more acceptable to employees as a form of informational support and can enhance the impact of role clarity and job competence on frontline employees’ taking charge behaviors.

## 1. Introduction

As organizations face an increasingly uncertain and dynamic environment, their survival and development depend on more than just employees’ in-role behaviors in accordance with their responsibilities, processes, and norms (Banks et al., 2023). Organizations expect their employees to make more proactive contributions by consistently innovating and adapting, which in turn enhances the agility and productivity of the organization (Dysvik et al., 2016; Almustafa et al., 2025). This is especially crucial in the hospitality industry, where frontline employees often contact customers from various regions, each with unique expectations and requirements. Employees often need to go beyond their job responsibilities and adapt to specific situations to provide instant responses to meet customer needs (Yan et al., 2023; Mansoor et al., 2025). With the growing emphasis on service differentiation and personalization, hotels expect their employees to engage actively and demonstrate greater flexibility with their services. Front-line employees are encouraged to identify potential opportunities in their workplace and exhibit proactive behaviors accordingly (PJ et al., 2023). Taking charge behavior, a positive extra-role behavior, entails employees taking spontaneous initiative to invest additional effort in the workplace and assume extra responsibility beyond their assigned tasks, and it has received widespread attention from both academia and practice (Morrison & Phelps, 1999; Bormann, 2024; Kong et al., 2025).

Taking charge behavior is different from many extra-role behaviors, such as pro-social or citizenship behaviors, which focus on the employee’s return on the organization’s support. It is more about employees anticipating future problems and driving change (Morrison & Phelps, 1999). Organizational sustainability benefits from employees’ voluntary initiatives to refine and optimize work processes and operational methods (T.-Y. Kim et al., 2015). Existing research highlights organizational and leadership factors as key antecedents of employees’ taking charge behaviors. Sustainable HRM practices (Li et al., 2019; Kumar et al., 2022) and organizational change (Homberg et al., 2019; Lyu et al., 2023) can motivate employees to proactively adapt and assume responsibility. Leadership styles (e.g., empowering, authentic, inclusive) further foster such behaviors by strengthening organizational identity and positive emotions (Li et al., 2019; S. Guo & Yang, 2024; Liu et al., 2024). By engaging in taking charge behavior, employees experience greater job satisfaction and career development while also contributing to improved individual, team, and organizational performance (Guzman et al., 2024; L. Zhou et al., 2024).

However, current studies focus mainly on how to increase employees’ motivation to take on additional responsibilities, with limited examination of the formation and maintenance of taking charge behavior from a comprehensive perspective. Drawing on Proactive Motivation Theory, this study aims to explore the impact of role clarity and job competence on frontline employees’ taking charge behaviors through a paired survey of 352 front-line employees and their supervisors in high-star hotels located in the Greater Bay Area of China. As indicated in Proactive Motivation Theory, individual proactive behaviors are characterized as motivated, conscious, and goal-directed actions. The formation and implementation of proactive goals hinge upon the premise that the individual “has the ability to do”, “has the reason to do”, and “has the energy to do” (Parker et al., 2010). Role clarity and job competence ensure that frontline employees “have the ability” to demonstrate taking charge behavior. When frontline employees have a clear understanding of their job requirements and ability to complete tasks, they can take on additional responsibilities with sufficient confidence (Chien et al., 2021; Satwika et al., 2025). They may more likely perceive themselves as valuable, competent, and significant contributors within their organization (S. L. Kim et al., 2023). Organization-based self-esteem strengthens their sense of responsibility and provides them “reasons “to make extra contributions. Supervisor developmental feedback emphasizes the importance of supervisors providing helpful and valuable information to their subordinates that can help them learn and grow in their work (C. Wang et al., 2023). It provides the “energy” to reinforce the impact of role clarity and job competence on frontline employees’ taking charge behaviors in the hospitality industry. By examining the mediating role of organization-based self-esteem and the moderating role of supervisor developmental feedback, the possible contribution of this study is to explain the formation mechanisms and boundary conditions of employees’ taking charge behaviors based on Proactive Motivation Theory. It clarifies that employees’ proactive extra-role responsibilities require not only willingness but also a comprehensive understanding of the formation and reinforcement of taking charge behaviors in terms of “ability”, “reason”, and “energy”. This is an empirical test and content expansion of taking charge behavior studies. Meanwhile, the findings will help the hospitality industry to better encourage and promote frontline employees to take the initiative to undertake extra duties, thus improving the overall service quality.

This paper is organized as follows. First, we provide a literature review and develop research hypotheses based on Proactive Motivation Theory. Next, we describe the research methodology including the study design, data collection procedures, and measurement instruments. Following this, we employ statistical analysis using SPSS 26 and Mplus 8.3 to test the proposed hypotheses and examine the relationships among the variables. Finally, we conclude the study by discussing the theoretical contributions and practical implications of the findings, as well as outlining potential directions for future research.

## 2. Theoretical Basis and Research Hypotheses

### 2.1. Employees’ Taking Charge Behaviors

Organizations expect employees not only to be competent in their own jobs but also to proactively take on responsibilities beyond their roles to improve personal and organizational performance (Morrison & Phelps, 1999). Taking charge behavior is not a formally required job duty by the organization, but rather an employee-initiated behavior that involves the initiation and implementation of positive change (Guzman et al., 2024). It is inherently change-oriented and demonstrates an employee’s efforts to challenge the status quo. After evaluating the expected costs and benefits, employees are willing to suggest changes when the organization is not functioning well (Dysvik et al., 2016). Thus, taking charge involves not only raising problems but also going further by offering suggestions on how to address those issues or opportunities (Kong et al., 2025). Employees can help organizations achieve desired goals by proposing appropriate strategies, procedures, and methods to solve existing problems (T.-Y. Kim et al., 2015). Taking charge behavior is considered a constructive effort voluntarily taken by employees to achieve improvement in the organization’s functioning (Grant et al., 2009). Employees who adopt taking charge behaviors may receive more support from their colleagues, which is also conducive to their career resilience and development (L. Zhou et al., 2024). In the hospitality industry, employees with high taking charge behaviors are more likely to proactively identify and resolve potential problems, guaranteeing service quality and minimizing the risk of customer complaints. It directly affects the reputation and competitiveness of the hotel (Chien et al., 2021). In the light of Proactive Motivation Theory, an individual’s proactive behavior is a conscious, goal-oriented action aimed at changing the current situation. To achieve proactive goals, individuals need to possess the necessary “abilities”, “motivations”, and “energy” (Parker et al., 2010). Taking charge behavior requires not only passion and willingness but also adequate knowledge, skills, and abilities, as well as a clear understanding of job requirements, work processes, and responsibility boundaries.

### 2.2. Role Clarity

Role clarity is considered an employee’s expectation of his or her position in the organization as well as a full understanding and awareness of the job role (Kahn et al., 1964). It can provide relevant information for employees to adequately perform their job duties, including work expectations, job-specific tasks, and feedback on work results (Lee et al., 2024). Employees need to clarify the job content and objectives, as well as understand the specific responsibilities and authority to complete the job. A lack of relevant information can prevent employees from properly understanding what is expected of them by their job roles, which can lead to role ambiguity and discourage employees from taking ownership of their work tasks. This is also detrimental to the achievement of work goals (Andriansyah et al., 2023). Role clarity can enhance individuals’ perceived fairness and identification with the organization. A high level of role clarity makes employees feel more enjoyable at work, helps them to better cope with changing environments, and increases individual engagement and innovation (Bernuzzi et al., 2023; Fang et al., 2025). They usually experience higher job satisfaction and organizational commitment and have a lower intention to leave (Ul-Hassan et al., 2021). Conversely, role ambiguity is often accompanied by negative outcomes such as higher job stress, work burnout, and withdrawal behaviors (Zhang et al., 2023). Proactive Motivation Theory states that individuals exhibit proactive behaviors through three motivational pathways: ability, reasons, and energy. The prerequisite for employees to demonstrate positive extra-role behaviors is that they need to be aware of the requirements of their duties (Parker et al., 2010). Role clarity holds substantial value by positively shaping employee behaviors while alleviating the psychological strain associated with role conflict and ambiguity (Abraham et al., 2022). Role clarity provides employees with adequate information to make behavioral decisions, which can reduce decision-making uncertainty and decrease behavioral risk. Meanwhile, it can increase an individual’s confidence to expand work scope and take on more responsibilities (Yadav & Rangnekar, 2015). Hypotheses 1 is proposed:

**H1.**  *Role clarity positively influences frontline employees’ taking charge behaviors*.

### 2.3. Job Competence

When employees clearly understand the requirements of the job, they evaluate their abilities and determine if they are capable of doing the job. At this point, job competencies which describe knowledge, abilities, skills, or traits that are linked to a job or job performance will reflect an employee’s capacity to successfully perform a job task (Fadhli et al., 2022; Dokoohaki et al., 2024). Competencies can help to differentiate between high-performing and average-performing employees. Individuals with high job competence can better accomplish the goals set by the organization (Swanson et al., 2020). Organizational support and career management activities (including training, mentoring, and socialization opportunities) in the workplace play an important role in improving career competence (Jo et al., 2024). Social support, autonomy, development opportunities, and job resources also have a positive impact on employees’ job competence and can enhance their engagement (Akkermans et al., 2013). Employees with high job competence often exhibit high levels of career identity and commitment. They can set appropriate career goals and excel in building social networks (Presti et al., 2022; Kett et al., 2023). Job competence has a positive impact on an individual’s subjective career success by enhancing career autonomy and reducing career insecurity (Presti et al., 2022). As indicated in Proactive Motivation Theory, “ability” is a prerequisite for individuals to demonstrate proactive behavior (Parker et al., 2010). Job competence ensures that the employee has sufficient knowledge, abilities, and skills to complete job requirements and meet organizational expectations (Swanson et al., 2020). Frontline employees in the hospitality industry are often faced with high levels of flexibility and uncertainty in their work. It is only when employees have a clear understanding of their job tasks, responsibilities, and goals, along with possessing the necessary competence, that they will consider taking on additional responsibilities (Parker et al., 2010; Satwika et al., 2025). These behaviors go beyond the basic job requirements and contribute to the overall success of the organization (Grant et al., 2009; Guzman et al., 2024). Hypothesis 2 is proposed.

**H2.**  *Job competence positively influences frontline employees’ taking charge behaviors*.

### 2.4. Organization-Based Self-Esteem

In the workplace, self-confidence is often reflected through organization-based self-esteem, which refers to an employee’s self-perception and self-evaluation of their contribution, significance, and influence within the organization (Pierce et al., 1989). Compared to individual self-esteem, organization-based self-esteem is a better predictor of employee performance and behaviors. Individuals with high organization-based self-esteem hold the belief that they can fulfill organizational needs by actively engaging in roles within the organization that align with their self-worth (J. Kim et al., 2024). Consequently, they perceive themselves as significant, purposeful, competent, and valuable contributors to the organization (Gordon & Hood, 2021). They usually have a stronger sense of responsibility and are more willing to participate in activities that are meaningful to organizational development (Dhir et al., 2024). To maintain a positive image of themselves in the organization, they more readily display voice, sustainable safety, helping, and other positive extra-role behaviors. Conversely, individuals with low organization-based self-esteem are more likely to choose to remain silent about many concerns and worries (Hur et al., 2022).

In line with Proactive Motivation Theory, with the appropriate “abilities”, employees also need “reasons” to generate proactive behaviors (Parker et al., 2010). When they believe they can take on extra responsibilities and can contribute to the organization, they have the willingness to respond to change proactively. Work is an important evaluation source for individuals to gain importance and value in an organization. When employees’ competencies match the job requirements, their self-efficacy increases. Clarity about job goals and job requirements can enhance an individual’s confidence in completing the job and confirms his or her value in the organization (Bernuzzi et al., 2023). For employees with high achievement motivation, adequate competence and job autonomy can stimulate a sense of meaning at work and generate positive psychological experiences, which are often accompanied by higher organization-based self-esteem (Pierce & Gardner, 2004; Hui et al., 2010). Employees’ evaluation of their competence and self-value as a member of the organization, as well as their identification with their performance, can serve as effective predictors of their work-related behaviors (Bowling et al., 2010). As indicated in Proactive Motivation Theory, organization-based self-esteem means that employees have “reasons” and confidence to perform additional work tasks. Compared to employees with low organization-based self-esteem, they are likely to have more positive work attitudes and exhibit more extra-role behaviors (Pierce et al., 1989). Thus, employees with higher job competence and role clarity are more likely to recognize their value in an organization, which can stimulate positive feelings and willingness to take on extra responsibility and put in more effort. They may show more taking charge behaviors. Therefore, research Hypotheses 3 and 4 are proposed.

**H3.**  *Organization-based self-esteem mediates the relationship between role clarity and frontline employees’ taking charge behaviors*.

**H4.**  *Organization-based self-esteem mediates the relationship between job competence and frontline employees’ taking charge behaviors*.

### 2.5. Supervisor Developmental Feedback

Employees need to know whether their behavior is what the organization expects and whether their extra efforts are yielding positive outcomes. Developmental feedback from supervisors can play a crucial role in sustaining and reinforcing employees’ proactive behaviors (C. Wang et al., 2023). Unlike traditional feedback, which is more concerned with performance standards, supervisor developmental feedback provides subordinates with helpful and valuable information that assists them in learning, developing, and advancing in their jobs (J. Zhou, 2003). It does not obligatorily set strict work requirements or plans for employees but rather provides information related to an individual’s future career development in a casual and relaxed atmosphere, which can enhance intrinsic motivation by guiding employees to integrate into their work (J. Zhou, 2003; C. Wang et al., 2023). The primary objective of supervisor developmental feedback is to concentrate on the future growth and development of employees. It involves providing constructive and developmental information to assist employees in acquiring new knowledge and skills, as well as helping them recognize and leverage their strengths (Sibunruang & Kawai, 2024). Feedback is given in the form of encouragement rather than appraisal, and no pressure is placed on employees for specific results, which focuses on the employee’s career growth and provides all possible assistance (Desa & Ding, 2016). Developmental feedback from supervisors is positive and informative feedback that can build trust between employees and their leaders, contribute to supervisor–subordinate exchange relationships, and promote the improvement of employees’ professional skills and job performance (Y. Guo et al., 2020). It conveys the organization’s support and encouragement for employees’ future development, reduces their anxiety, and positively influences employees’ creativity by improving their related skills and intrinsic motivation (Bak, 2020; Udahemuka et al., 2024). Developmental feedback from supervisors also can stimulate employees’ work interest, increase their organizational commitment, and generate positive emotions from work (Y. Guo et al., 2020; Sibunruang & Kawai, 2024).

Taking charge reflects employees’ proactive approach to change, where they actively seek ways to enhance both personal and organizational performance (Moon et al., 2008). According to Proactive Motivation Theory, it also requires enough “energy” to maintain this positive extra-role behavior (Parker et al., 2010). Adequate job competency and clear job guidelines are the foundation for frontline employees to fulfill their job duties (Parker et al., 2010). As a spontaneous extra-role behavior, taking charge behavior embodies the employee’s proactive effort to improve work processes and methods (Moon et al., 2008). It places higher demands on employee competence and role clarity, especially in a changing external environment where the job content and the organization’s expectations are constantly evolving. Employees need to continually strengthen themselves to better meet the needs of organizational development (Solberg et al., 2022). Competence and role clarity can enhance an individual’s sense of self-worth in an organization. In addition, organization-based self-esteem, a positive psychological resource, can stimulate employees’ enthusiasm and sense of responsibility (M. Wang et al., 2022; Hur et al., 2022). Employees with high organization-based self-esteem perceive themselves as loyal and contributing members of the organization and are more likely to have a positive self-concept (Pierce & Gardner, 2004). They are also more proactive in their work and usually believe they can play an important role in the organization. During the process, developmental feedback from supervisors acts as a source of energy and sustenance, supporting frontline employees to continually accept challenges and demonstrate proactive behaviors. As indicated in Proactive Motivation Theory, individuals need sufficient “energy” to stimulate and maintain their proactive behavior (Parker et al., 2010). When an employee’s immediate supervisor offers valuable information about future learning and development, it not only increases the likelihood of acceptance and trust from subordinates but also enhances their overall positive work experience (Kilroy et al., 2023; C. Wang et al., 2023). Through the guidance of supervisors, employees take the initiative to adjust their behaviors and attitudes, and their skills are enhanced to better cope with the challenges of the changing environment (Udahemuka et al., 2024). Thus, when receiving a higher level of developmental feedback from their supervisors, frontline employees with job competence and role clarity are more likely to feel higher organization-based self-esteem and provide more flexible services. They have gained the “energy” to inspire more taking charge behaviors. Supervisor developmental feedback is a boundary condition for the influence of role clarity and job competence on frontline employees’ taking charge behaviors through organization-based self-esteem. Therefore, research hypotheses 5 and 6 are proposed.

**H5.**  *Supervisor developmental feedback positively moderates the indirect effect of role clarity on frontline employees’ taking charge behaviors through organization-based self-esteem*.

**H6.**  *Supervisor developmental feedback positively moderates the indirect effect of job competence on frontline employees’ taking charge behaviors through organization-based self-esteem*.

### 2.6. The Current Study

**Figure 1:**
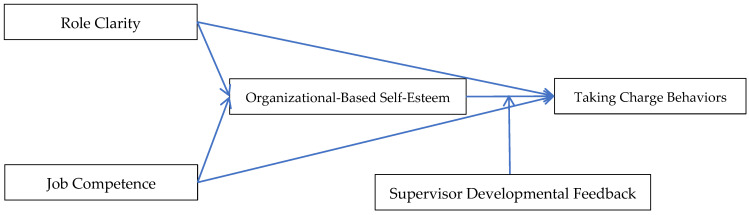
Theoretical model.

This study aims to examine the influence mechanisms of job competence and role clarity on the employees’ taking charge behaviors and the moderating effect of supervisor developmental feedback. The research framework is shown in Figure 1: role clarity and job competence influence frontline employees’ taking charge behaviors through organization-based self-esteem. Supervisor developmental feedback moderates the indirect effect of role clarity and job competence on frontline employees’ taking charge behaviors through organization-based self-esteem.

## 3. Method

### 3.1. Procedure

With the help of our DBA and EMBA students, we obtained support from the HR departments of 13 high-star hotels located in the Greater Bay Area of China. As suggested by Podsakoff et al. (2003), procedure control was used to reduce common method bias. Data were collected from frontline employees and their direct supervisors together. The online questionnaire was distributed in three waves through WJX (a widely used online survey platform in China). Time 1: Convenience sampling was used to collect information about job competence and role clarity of frontline employees in hotels. Due to limitations in research conditions, respondents were selected to participate in the survey based on personal relationships and convenience principles (Staller, 2021). Each respondent was asked to fill in the last four digits of their phone number as the questionnaire code. Time 2: Data on taking charge behaviors of frontline employees in hotels were collected from their supervisors with the help of the HR department. Time 3: Data on organization-based self-esteem, supervisor developmental feedback, and demographic information were collected from frontline employees in hotels by number matching. Respondents were asked to fill in the last four digits of their phone number again, which was used to match the questionnaire with the first survey. In addition, to reduce response bias and acquaintance bias, respondents were clearly informed about the study’s purpose and the research procedures. The questionnaire was completed voluntarily, and respondents were allowed to terminate the survey at any time. The respondents were guaranteed that all their identification would be removed after the questionnaire was matched. The data obtained from the survey will be kept by the researchers and only used for academic research (Bryan et al., 2013; Teh et al., 2023).

### 3.2. Ethics

The study was reviewed by the Research Ethics Committee of BNU-HKBU United International College. All methods in the study were performed following the Declaration of Helsinki. A total of 480 sets of questionnaires were distributed, 379 sets were recovered, and 352 sets of questionnaires were valid. The effective recovery rate was 73.3%.

### 3.3. Participants

All respondents were from high-star hotels in the Greater Bay Area of China. The valid sample for this study included 352 employees and 114 supervisors. Employee Sample: In terms of gender, males accounted for 47.4% (167) of the respondents, females accounted for 52.6% (185) of the respondents, and the gender ratio was relatively balanced. In terms of age, most respondents were 21–30 years old, accounting for 55.4% (195) of the total sample. The respondents less than 20 years old accounted for 17.6% (62) of the respondents, and respondents more than 30 years old accounted for 27.0% (95) of the respondents. About half of the respondents had received higher education, accounting for 58.2% (205) of the total sample. The respondents were mainly from Lobby Department (16.8%), Room Department (33.05%), Catering Department (21.3%), and Clubhouse (19.3%). Most of the respondents had 3–5 years of seniority (51.7%), 19.9% of the respondents had less than 3 years of seniority, and 28.4.9% of the respondents had more than 5 years of seniority. Supervisor Sample: In terms of gender, males accounted for 62.3% (71) of the respondents, and females accounted for 37.3.6% (43) of the respondents; 7% (8) of supervisors were less than 20 years old, 42.1% (48) of supervisors were between 21 and 30 years old, 45.6% (52) of supervisors were between 31 and 40 years old, and 5.3% (6) of supervisors were more than 41 years old. Most of the respondents had received higher education, accounting for 60.5% (69) of the total sample.

### 3.4. Measurement

The survey was conducted with Likert 5-point scales from “complete disagreement” to “complete agreement”.

Role Clarity: Role clarity refers to an employee’s comprehensive understanding of their job responsibilities and tasks (Kahn et al., 1964). It was measured using the scale developed by Rizzo et al. (1970), which has 3 items, and the Cronbach’s alpha coefficient of this scale in the study was 0.865. The specific item is as follows: “I know exactly what my position expects on my role”.

Job competence: Job competencies refer to the knowledge, abilities, skills, motivations, or traits that are directly related to a specific job or job performance, which are indicative of an employee’s ability to effectively carry out job tasks, fulfill assigned roles, or hold a particular position (Fadhli et al., 2022). It was measured using the scale developed by Jokinen et al. (2008), which has 7 items, and the Cronbach’s alpha coefficient of this scale in the study was 0.864. The specific item is as follows: “I have job-related skills and knowledge”.

Organization-based self-esteem: Organization-based self-esteem (OBSE) refers to specific self-esteem based on the organizational level, which is an employee’s self-perception and self-assessment of his or her contribution, importance, and influence in the organization (Pierce et al., 1989). It was measured using the scale developed by Pierce et al. (1989). which has 10 items, and the Cronbach’s alpha coefficient of this scale in the study was 0.953. The specific item is as follows: “I’m very important in this hotel”.

Taking charge behavior: Taking charge behavior is a voluntary behavior that aims at improving the way individuals and organizations work, which is inherently change-oriented and demonstrates an employee’s efforts to challenge the status quo (McAllister et al., 2007). It was measured using the scale developed by Morrison and Phelps (1999), which has 10 items, and the Cronbach’s alpha coefficient of this scale in the study is 0.915. The specific item is as follows: “The subordinate frequently attempts to use improved procedures to get the job done”.

Supervisor developmental feedback: Supervisor developmental feedback describes the extent to which supervisors provide their subordinates with helpful or valuable information that assists them in learning, developing, and advancing in their jobs (J. Zhou, 2003). It was measured using the scale developed by J. Zhou (2003), which has 3 items, and the Cronbach’s alpha coefficient of this scale in the study was 0.759. The specific item is as follows: ”While giving me feedback, my supervisor focuses on helping me to learn and improve”.

Control variables: Demographic characteristics are widely recognized as potential factors that can influence employees’ proactive behaviors. Individuals belonging to specific demographic groups may demonstrate varying levels of proactive assumption of responsibilities (Liang et al., 2012; Satwika et al., 2025). Based on a review of existing studies, gender, age, education, position, and seniority, which have the potential to impact employees’ taking charge behaviors, were considered as control variables (Morrison et al., 2011; Mussner et al., 2017; Dysvik et al., 2016; Zheng et al., 2017). Independent-samples *t*-test and ANOVA test showed that there was no significant difference between the respondents’ organization-based self-esteem and taking charge behaviors in terms of gender, age, education, and seniority, except for position. Position needs to be controlled in subsequent statistical analyses (Cinelli et al., 2024). Given that it is a categorical variable, we transformed it into a dummy variable for correlation and hierarchical regression analysis as follows: 0 = others; 1 = Lobby Department; 2 = Catering Department; 3 = Room Department; 4 = Clubhouse.

### 3.5. Data Analysis

The data analysis and hypotheses testing were performed using SPSS 26 and Mplus 8.3 software. The analytical procedure consisted of four main steps. First, to control the effect of common method bias and assess scale validity, we initially conducted Harman’s single-factor test using SPSS 26, followed by confirmatory factor analysis (CFA) performed in Mplus 8.3 (Podsakoff et al., 2003). In Harman single-factor test, the variance explained by the first factor was 34.75%, which is much lower than the threshold level of 50%. The common method bias in our study was not significant. Subsequently, SPSS 26 was employed to compute descriptive statistics and correlations among study variables. Each variable was measured using a 5-point Likert scale and did not violate the normal distribution based on kurtosis and skewness tests (Kline, 2020). Therefore, Pearson correlation analysis was used in this study. Third, hierarchical regression analysis was performed using SPSS 26 to examine the mediating effect of organization-based self-esteem and the moderating effect of supervisor developmental feedback, as suggested by Baron and Kenny (1986). The direct effect and indirect impact of role clarity and job competence on frontline employees’ taking charge behaviors were examined through bootstrapping tests. Finally, Mplus 8.3 was utilized to test the moderating mediation effect. To further clarify this moderating influence, we compared the conditional indirect effects at high (Mean + 1SD) and low (Mean − 1SD) levels of supervisor developmental feedback.

## 4. Results

### 4.1. Confirmatory Factor Analysis

The confirmatory factor analysis results show that the overall fit of the five-factor model (role clarity, job competence, organization-based self-esteem, taking charge behavior, developmental feedback) is better (χ^2^/*df* = 2.343, RMSEA = 0.062, CFI = 0.945, TLI = 0.937, SRMR = 0.056) than that of other alternative structure models. The model fit of the single-factor model is far from acceptable (χ^2^/*df* = 16.090, RMSEA = 0.207, CFI = 0.342 TLI = 0.298 SRMR = 0.1126). Therefore, the five-factor model demonstrates acceptable model fit for the data, indicating satisfactory discriminant validity for this study.

### 4.2. Descriptive Statistics

The results of the descriptive statistical analysis are shown in Table 1. Means, standard deviations, medians, and ranges are reported for role clarity, job competence, organization-based self-esteem, taking charge behavior, and developmental feedback.

### 4.3. Correlation Analysis

Pearson correlation analysis results are shown in Table 2. When controlling for the effects of employee gender, age, education, position, and seniority, role clarity has a positive correlation with organization-based self-esteem and taking charge behavior; job competence has a positive correlation with organization-based self-esteem and taking charge behavior; and organization-based self-esteem has a positive correlation with taking charge behavior.

### 4.4. Hypothesis Testing

Hypothesis 1 and 2 proposed that role clarity and job competence are positively related to frontline employees’ taking charge behaviors. The results in Table 3 indicate that the direct effect of role clarity on frontline employees’ taking charge behaviors is significant; the direct effect of job competence on frontline employees’ taking charge behaviors is significant. Research hypotheses 1 and 2 are supported.

Hypothesis 3 proposed that organization-based self-esteem mediates the relationship between role clarity and frontline employees’ taking charge behaviors. The results in Table 3 indicate that the indirect effect of role clarity on frontline employees’ taking charge behaviors through organization-based self-esteem is significant. Organization-based self-esteem has a partial mediating role in the relationship between role clarity and frontline employees’ taking charge behaviors. Hypothesis 3 is supported.

Hypothesis 4 proposed that organization-based self-esteem mediates the relationship between job competence and frontline employees’ taking charge behaviors. The results in Table 4 indicate that the indirect effect of role clarity on frontline employees’ taking charge behavior through organization-based self-esteem is significant. Organization-based self-esteem has a partial mediating role in the relationship between job competence and frontline employees’ taking charge behaviors. Hypothesis 4 is supported.

Hypothesis 5 proposed that supervisor developmental feedback positively moderates the indirect effect of role clarity on frontline employees’ taking charge behavior through organization-based self-esteem, such that the relationship is stronger when supervisor developmental feedback is higher (vs. low). The results in Table 5 indicate that the moderating effect is significant. The indirect effect of role clarity on frontline employees’ taking charge behaviors through organization-based self-esteem is significant when supervisor developmental feedback is high. Hypothesis 5 is supported.

Hypothesis 6 proposed that supervisor developmental feedback positively moderates the indirect effect of job competence on frontline employees’ taking charge behaviors through organization-based self-esteem, such that the relationship is stronger when supervisor developmental feedback is higher (vs. low). Results in Table 5 indicate that the moderating effect is significant. The indirect effect of job competence on frontline employees’ taking charge behaviors through organization-based self-esteem is significant only when supervisor developmental feedback is high. Hypothesis 6 is supported.

## 5. Discussion

Today’s organizations are facing volatile, uncertain, complex, and ambiguous external environments (Scheibel et al., 2025). Especially after the COVID-19 epidemic, although the hospitality industry is rapidly recovering, it is also confronted with a high degree of uncertainty and change (Almustafa et al., 2025). The requirements and expectations of hotel customers have changed significantly, placing higher requirements on the whole service industry. Traditional standard processes may no longer be able to meet the current needs of customers. To increase customer satisfaction and, at the same time, help hotels cope with the fierce industry competition (PJ et al., 2023), the value of positive extra-role behaviors of frontline employees is becoming increasingly important (S. L. Kim et al., 2023). Organizations expect employees to take the initiative to improve their behaviors and services based on customer requirements beyond completing their work and to make more rationalized suggestions regarding work procedures (S. Guo & Yang, 2024). Based on Proactive Motivation Theory, this study investigated the mechanisms underlying frontline employees’ taking charge behaviors in the hospitality industry. All hypotheses were supported.

### 5.1. Theoretical Implications

The findings indicated that organization-based self-esteem mediates the relationship between role clarity, job competence, and frontline employees’ taking charge behaviors. Role clarity and job competence can enhance frontline employees’ taking charge behaviors by increasing their organization-based self-esteem. This shows that clear role requirements and adequate competencies can positively influence employees’ positive emotions and behaviors, which is consistent with previous studies (Presti et al., 2022; Kett et al., 2023; Mansoor et al., 2025). When employees have a thorough understanding of the job scope and performance requirements, they are less likely to be confused at work. Competence ensures that they have the confidence to perform their duties as well as gain fulfillment and achievement from their work (Kett et al., 2023; Almustafa et al., 2025). Role clarity and job competence are necessary for frontline employees to develop their taking charge behaviors. This study also verified the moderating effect of supervisor developmental feedback. It moderates the indirect effect of role clarity and job competence on frontline employees’ taking charge behaviors through organization-based self-esteem. When obtaining more developmental feedback from supervisors, the influence of front-line employee role clarity and job competence on their taking charge behaviors through organization-based self-esteem is stronger. Existing research has adequately demonstrated that the interaction between superiors and subordinates can result in a healthy exchange relationship (Gordon & Hood, 2021). The feedback from leaders can encourage employees to properly evaluate and adjust their behaviors in the workplace, which in turn can help them achieve individual performance goals (Su et al., 2019; Bak, 2020). Unlike traditional performance-based feedback, developmental feedback emphasizes that supervisors provide valuable information for subordinates’ development (Sibunruang & Kawai, 2024). It serves as a form of information exchange and communication that contributes to employee growth and development (J. Zhou, 2003). It is a boundary condition for the influence of role clarity and job competence on frontline employees’ taking charge behaviors through organization-based self-esteem. Unlike previous studies that have only considered one perspective, this study combines three viewpoints—job characteristics, skill requirements, and leadership feedback—to explain how taking charge behavior is developed and reinforced. It helps to gain a comprehensive understanding of employees’ positive extra-role behaviors in the workplace.

Proactive Motivation Theory is reinforced through an examination of the impact mechanisms and limiting factors of role clarity and job competence on frontline employees’ proactive behaviors. An individual’s proactive behavior is goal-focused and initiated to change the status quo (Xu et al., 2022). Achieving proactive goals requires that individuals “have abilities”, “have reasons”, and “have energies” (Parker et al., 2010). While employee taking charge behavior, which involves voluntarily seeking to improve work processes, has garnered significant attention, existing research predominantly examines its emergence from the perspective of employees’ willingness (Li et al., 2019). Organizations do not just need to provide employees with “reasons” for taking charge, they need to make sure employees have sufficient capacities. For many employees, the problem they may face is not unwillingness but rather that they are not competent enough to know how to perform extra-role behaviors expected by the organization. Furthermore, when individuals encounter ambiguity in their job roles, they may struggle to comprehend the expectations and assessment criteria associated with their positions (Zhang et al., 2023). In particular, the failure to accurately define the scope of their responsibilities will limit the likelihood that they will make proactive changes and positive improvements (Andriansyah et al., 2023). Role clarity and job competence, as “abilities” factors, are prerequisites for the formation of positive extra-role behaviors. Clear role requirements and high levels of job competence enhance the individual’s perceived value to the organization, which increases their confidence and willingness to take the initiative to provide extra services and improve work processes. “Reasons” are met as the second condition that facilitates employees to take on additional responsibilities. Taking charge behaviors require that employees go beyond their duties and put in extra effort. This also means that enough “energy” is needed to keep up the enthusiasm for extra work (Moon et al., 2008). In general, developmental information provided by supervisors is more acceptable to employees than information provided by other sources, which can give more help to subordinates (J. Zhou, 2003; Liu et al., 2024). Supervisor development feedback was confirmed to be an “energy” factor that enhances the impact of role clarity and job competence on frontline employees’ taking charge behaviors. It enables employees to constantly reflect on themselves, which inspires their job confidence and enthusiasm for active learning. Employees can strengthen the knowledge, skills, and abilities needed for their jobs through information support from their supervisors (C. Wang et al., 2023). Simultaneously, employees can adjust their perception of job responsibilities to align more effectively with the organization’s expectations by engaging in information exchange with their supervisors. Through collaborative efforts between frontline staff and supervisors, employees can improve service flexibility and more effectively address customer needs through their proactive behaviors. Proactive Motivation Theory provides solid theoretical support for examining the taking charge behaviors of frontline employees. Under the framework of proactive motivation, a thorough investigation into employee taking charge behavior extends the current research on extra-role behaviors. This not only enhances the comprehension of employee behavior but also delivers crucial validation and guidance for the practical application of Proactive Motivation Theory.

### 5.2. Practical Implications

Due to the need for frontline employees to face customers directly and deal with many unexpected situations, the hospitality industry always has high expectations for their taking charge behavior (Yan et al., 2023). In a dynamic competitive environment, employees face constantly changing job requirements and sudden work tasks. Merely fulfilling the prescribed responsibilities cannot guarantee the high-quality completion of their work objectives (Ghlichlee & Bayat, 2021). It is already a common pursuit of all organizations to motivate employees to demonstrate high initiative and innovation in the workplace. Organizations not only in the hospitality industry but also in different industries need to take effective measures to encourage employees to show more taking charge behaviors while performing their duties.

Employees should be clearly informed of the requirements, processes, duties, and responsibilities of their work. Any ambiguity in job roles enhances employees’ perceived insecurity (Andriansyah et al., 2023). Emotionally, they will perceive themselves as unimportant to the organization, lowering their sense of belonging to the organization, which will reduce their willingness to display positive extra-role behaviors. Practically, role ambiguity prevents employees from accurately defining the scope of their responsibilities. To minimize risk, they reduce their effort and even avoid performing their duties (Ahmad et al., 2021). Therefore, it is important for employees to understand their job role requirements during orientation. Organizations need to provide a clear employee handbook when new employees are onboarded. Supervisors also need to maintain ongoing communication with employees about the organization’s expectations. Especially for frontline staff, managers need to provide instant feedback on customer complaints, suggestions, and satisfaction surveys to help them adjust their working methods and improve service quality.

Organizations need to ensure that employees have the competence to perform their jobs and fulfill additional responsibilities. Job competence is the basis of in-role and extra-role behaviors (Fadhli et al., 2022). Many employees may be willing to contribute extra efforts to organizational development, but if they do not have the relevant knowledge, skills, or abilities, they may face communication barriers with customers or colleagues (Kett et al., 2023). Even if they are prepared to respond to changing job demands, their incompetence may result in unnecessary troubles for other colleagues or the department. Specifically, organizations should promote a culture of lifelong learning within the organization, encouraging employees to be aware of and respond positively to changes in the environment. Organizations can improve employee competency through training and career development programs. Mentorship and internal sharing also help employees to update their competence and service concepts.

Organizations should strive to enhance employees’ positive experiences and organization-based self-esteem in the workplace. When employees face sudden or highly challenging tasks, the lack of timely support and encouragement from the organization may result in a growing sense of detachment and disengagement. When work is considered only as a means of earning a living, a range of positive extra-role behaviors, such as taking charge behaviors, voice, and helping will diminish and disappear. Organizations need to increase their employees’ confidence in their jobs by improving their competence. By collaborating with employees to develop career plans and providing career support, organizations can enhance employees’ sense of belonging (Almustafa et al., 2025). Meanwhile, it is also important to be clear about the scope of employees’ duties and to emphasize the importance of their work to the organization. Organizations should set specific and measurable goals for employees and provide timely recognition and praise for their extra contributions through formal and informal channels.

Supervisors should take full advantage of the positive effects of developmental feedback. Although traditional performance feedback as a reward and punishment mechanism can provide employees with a clear understanding of their job performance, developmental feedback is more effective in stimulating their work enthusiasm and clarifying the direction of their efforts (Sibunruang & Kawai, 2024). Employees often obtain information through multiple channels and constantly adjust their working manner to better meet job requirements. Given the authority and extensive experience of managers, their feedback is of greater value to employees. Their encouragement and guidance not only inspire employees to work with greater effort but also make their suggestions more readily accepted by their subordinates (C. Wang et al., 2023). Supervisors should always communicate with their subordinates about their work objectives, which can reduce their work pressure. In addition, the supervisor should also give immediate encouragement to the subordinate’s extra effort. Especially when the subordinate encounters difficulties, they need to provide the necessary support and discuss the solution together. Developmental feedback reflects the positive interaction between supervisors and their subordinates (J. Zhou, 2003). It should be more recognized and promoted as a supplement to performance feedback.

## 6. Limitations and Future Study

The study has some limitations. First, the data collected in this study were all from hotels in the Greater Bay Area of China. Chinese culture values responsibility and rules. The antecedents and scenarios of taking charge behaviors in the Chinese context may differ significantly from those in other cultures, which may result in external validity issues for this study’s findings. Therefore, future research could consider comparing the mechanisms and boundary conditions that generate employees’ taking charge behaviors from a cross-cultural perspective.

Second, this study used convenience sampling to obtain data. Convenience sampling, a non-probability sampling method, has time and cost advantages that make it widely used in management research. However, as the researcher selects the most accessible individual or group as the sample, it may result in an under-representative sample that does not accurately reflect the characteristics of the target population (Staller, 2021). Probability sampling could be considered in future studies to obtain more representative data.

Finally, the study examined the influencing mechanism of job competence and job role clarity on hotel frontline employees’ taking charge behaviors. The occurrence of positive extra-role behavior requires not only “knowing how”, “knowing what” and “knowing why” but also “knowing who”, “knowing when”, and “knowing where” (Parker et al., 2010). Employees need to improve themselves to meet the changing environment and employment relationship. This is the basis for frontline staff to demonstrate taking charge behaviors and a prerequisite for highly resilient and personalized service in the hospitality industry. However, the formation and boundary conditions of employees’ taking charge behaviors are complicated. Future research can also further expand on the research perspectives and content, such as professional reputation, work motivation, exchange relationships with supervisors, customer feedback, organizational climate, and so on.

## 7. Conclusions

In line with Proactive Motivation Theory, this study examined the influence mechanisms and boundary conditions of role clarity and job competence on frontline employees’ taking charge behaviors. Through a paired survey of frontline employees and their supervisors in high-star hotels of the Greater Bay Area of China, the mediating role of organization-based self-esteem and the moderating role of supervisor developmental feedback were validated. The findings not only contribute to a comprehensive understanding of the formation of employees’ taking charge behavior from the perspectives of “ability”, “reason”, and “energy” but also provide practical guidance for organizations to effectively promote employees’ positive extra-role behavior.

## Figures and Tables

**Table 1:** Descriptive statistics (*n* = 352).

Variables	Mean	S.D.	Median	Ranges
Role Clarity	3.749	0.579	3.800	2.200–5.000
Job Competence	3.253	0.859	3.333	1.000–5.000
Organization-Based Self-Esteem	3.445	0.737	3.600	1.000–5.000
Taking Charge Behavior	3.348	0.793	3.300	1.440–5.000
Developmental Feedback	3.679	0.575	3.667	1.890–5.000

**Table 2:** Pearson correlation statistics (*n* = 352).

	1	2	3	4	5	6	7	8
1. Lobby								
2. Catering	−0.315 **							
3. Room	−0.233 **	−0.365 **						
4. Clubhouse	−0.220 **	−0.343 **	−0.255 **					
5. RC	−0.089	0.037	0.113 *	−0.051				
6. JC	−0.194 **	0.114 *	0.099	0.004	0.405 **			
7. OBSE	−0.153 **	0.043	0.110 *	−0.001	0.468 **	0.582 **		
8. TCB	−0.098	0.032	0.128 *	−0.011	0.365 **	0.460 **	0.401 **	
9. SDF	−0.152 **	0.039	0.174 **	−0.016	0.283 **	0.329 **	0.297 **	0.223 **

Note: ** *p* < 0.01, * *p* < 0.05; RC: role clarity, JC: job competence, OBSE: organization-based self-esteem, TCB: taking charge behavior, SDF: supervisor developmental feedback.

**Table 3:** Hierarchical regression (*n* = 352).

	DV: TCB			DV: OBSE		DV: TCB			
	M1	M2	M3	M4	M5	M6	M7	M8	M9
IV	β	β	β	β	β	β	β	β	β
Lobby	0.023	0.035	0.056	−0.083	−0.057	0.059	0.067	0.061	0.075
Catering	0.153	0.127	0.068	0.031	−0.043	0.118	0.077	0.114	0.123
Room	0.215 *	0.169 *	0.136	−0.1058	0.17	0.153 *	0.133 *	0.149	0.150
Clubhouse	0.101	0.101	0.058	0.030	−0.026	0.093	0.063	0.081	0.100
RC		0.350 ***		0.454 ***		0.220 ***			
JC			0.450 ***		0.574 ***		0.336 ***		
OBSE						0.285 ***	0.198 **	0.360 ***	0.352 ***
SDF								0.096	0.128 *
OBSE × SDF									0.202 ***
R^2^	0.029	0.149	0.221	0.233	0.343	0.211	0.246	0.182	0.221
F	6.504	12.128 ***	19.597 ***	20.995 ***	36.062 ***	15.418 ***	18.798 ***	12.783 ***	13.974 ***

Note: *** *p* < 0.001, ** *p* < 0.01, * *p* < 0.05; DV: dependent variable; IV: independent variable; RC: role clarity, JC: job competence, OBSE: organization-based self-esteem, TCB: taking charge behavior, SDF: supervisor developmental feedback, β: standardized regression coefficient.

**Table 4:** Direct effect and indirect effect (*n* = 352).

		Path	Estimate	S.E.	Est./S.E.	LLCI	ULCI
Path 1	Direct Effect	RC--TCB	0.321	0.069	4.629	0.185	0.450
	Indirect Effect	RC--OBSE-TCB	0.142	0.039	3.638	0.072	0.230
Path 2	Direct Effect	JC--TCB	0.335	0.071	4.690	0.200	0.484
	Indirect Effect	JC--OBSE-TCB	0.104	0.039	2.644	0.029	0.184

Note: RC: role clarity, JC: job competence, OBSE: organization-based self-esteem, TCB: taking charge behavior, LLCI: low level of 95% confidence interval, ULCI: upper level of 95% confidence interval.

**Table 5:** Moderated mediation effect (*n* = 352).

**Mediating Effect: RC → OBSE → TCB**						
**Moderator SDF**	**Effect**	**Coefficient**			**95%CI**	
		**S.E.**	**Est./S.E.**	***p***	**LLCI**	**ULCI**
High Level	0.244	0.050	4.46212	0.000	0.157	0.336
Low Level	0.054	0.041	1.315	0.189	−0.020	0.146
INDD	0.190	0.055	3.428	0.001	0.091	0.309
**Mediating Effect: JC → OBSE → TCB**						
**Moderator SDF**	**Effect**	**Coefficient**			**95%CI**	
		**S.E.**	**Est./S.E.**	***p***	**LLCI**	**ULCI**
High Level	0.191	0.038	4.953	0.000	0.121	0.271
Low Level	0.017	0.038	0.450	0.653	−0.051	0.100
INDD	0.174	0.042	4.099	0.000	0.096	0.263

Note: RC: role clarity, JC: job competence, OBSE: organization-based self-esteem, TCB: taking charge behavior, DF: developmental feedback. INDD: indirect difference, LLCI: low level of 95% confidence interval, ULCI: upper level of 95% confidence interval.

## Data Availability

The data presented in this study are available upon request from the corresponding author.

## Abbreviations

The following abbreviations are used in this manuscript:

RC	Role Clarity
JC	Job Competence
OBSE	Organization-Based Self-Esteem
TCB	Taking Charge Behavior
SDF	Supervisor Developmental Feedback.
